# Genome-Wide Association Studies for Comb Traits in Chickens

**DOI:** 10.1371/journal.pone.0159081

**Published:** 2016-07-18

**Authors:** Manman Shen, Liang Qu, Meng Ma, Taocun Dou, Jian Lu, Jun Guo, Yuping Hu, Guoqiang Yi, Jingwei Yuan, Congjiao Sun, Kehua Wang, Ning Yang

**Affiliations:** 1 Layer Breeding and Production, Jiangsu Institute of Poultry Science, Chinese Academy of Agricultural Science, Yangzhou, China; 2 National Engineering Laboratory for Animal Breeding and MOA Key Laboratory of Animal Genetics and Breeding, College of Animal Science and Technology, China Agricultural University, Beijing, China; Chinese Academy of Fishery Sciences, CHINA

## Abstract

The comb, as a secondary sexual character, is an important trait in chicken. Indicators of comb length (CL), comb height (CH), and comb weight (CW) are often selected in production. DNA-based marker-assisted selection could help chicken breeders to accelerate genetic improvement for comb or related economic characters by early selection. Although a number of quantitative trait loci (QTL) and candidate genes have been identified with advances in molecular genetics, candidate genes underlying comb traits are limited. The aim of the study was to use genome-wide association (GWA) studies by 600 K Affymetrix chicken SNP arrays to detect genes that are related to comb, using an F_2_ resource population. For all comb characters, comb exhibited high SNP-based heritability estimates (0.61–0.69). Chromosome 1 explained 20.80% genetic variance, while chromosome 4 explained 6.89%. Independent univariate genome-wide screens for each character identified 127, 197, and 268 novel significant SNPs with CL, CH, and CW, respectively. Three candidate genes, *VPS36*, *AR*, and *WNT11B*, were determined to have a plausible function in all comb characters. These genes are important to the initiation of follicle development, gonadal growth, and dermal development, respectively. The current study provides the first GWA analysis for comb traits. Identification of the genetic basis as well as promising candidate genes will help us understand the underlying genetic architecture of comb development and has practical significance in breeding programs for the selection of comb as an index for sexual maturity or reproduction.

## Introduction

It is important to know when sexual maturation occurs in poultry management, the comb provides reliable clues on the selection of reproductive physiology. Most studies reported on the comb are associated with sexual maturity [1]. Also, the comb has been reported to affect male social rank, mate choice, heat regulation [2], and is related to female egg production, fecundity, bone mass [3, 4], and also tarsus length [5]. Comb traits in chicken can be measured as comb length (CL), comb height (CH), and comb weight (CW).

The main comb function is related with sexual maturity. Studies show that the alleles that increase CW also decrease onset of sexual maturity [6] and the comb in female broiler breeders begins to grow larger as the pullets approach sexual maturity before laying [7]. Plasma estrogen concentration seems to increase concurrently with comb growth and age as the first egg advances [8]. Also, extra testosterone propionate that is related with sexual maturity hormones stimulates comb development in male fowls [9]. Moreover, lots of studies have revealed a positive relationship between paternal attractiveness and offspring quality [10–13]. Females may prefer more attractive males with larger combs to obtain better viability or attractiveness genes for their offspring [14]. While male preference for female ornaments, revealed that males preferentially allocated sperm to females with large sexual ornaments signaling superior maternal investment [4, 11].

Theoretically, the comb reflects female reproductive investment [15]. Compared to a small-combed, vasectomized mate, female birds produced more eggs when housed with a large-combed male [14]. Females with larger combs receive significantly more sperm from dominant males than subordinate males [4]. Calcium mobilizes into the eggshell more easily when hens have larger combs because more calcium is deposited in the diaphysis, which shows that CW was positively correlated with bone allocation [16]. For osteoporosis expressed later in life and were characteristics are difficult to evaluate, the comb shows great potential as a reproductive indicator [4].

The estimated heritability of the comb in chicken is high at hatching (0.76) whether with hormone stimulation or not [9], so it is necessary to adopt the comb as an important trait in chicken breeding. However, in comparison with other poultry science topics, such as egg production and egg weight, the comb has received little attention in the past 40 years. Therefore, there has been considerable research undertaken to develop genetic markers that can be used for marker-assisted selection. Recent studies show increasing use of molecular biology techniques to create both mechanistic and statistical descriptions of genotype-phenotype maps that have allowed greater understanding of quantitative traits [16–18].

There has been a steady growth in the application of genomic tools, which have created breakthrough strategies for the study of sexual ornaments [19]. Studies have found that quantitative trait loci (QTL) for CW were on chromosomes 1 and 3 [15] and a QTL for medullary bone was detected at the same locus that also affected comb weight on chromosome 3, the hydroxyacid oxidase (*HAO1*) gene and bone morphogenetic protein 2 (*BMP2*) were adjacent to it [16]. These works throw light on the genetic basis of comb but were not able to explain the detailed genetic architecture involved in the comb growth. Nevertheless, larger population studies on the quantitative traits are very scare, which encouraged us to conduct in-depth studies of the comb. Recently, the precision of gene-level mapping of Genome-wide association (GWA) studies have been employed to reveal the associations between genomic loci and phenotypes with single nucleotide polymorphism (SNP) arrays in chicken [20]. With the development and availability of the 600 K Affymetrix Chicken SNP array, the candidate genomic segments and pinpointing several dominating causal variants could be narrowed down [21], eg. heat tolerance [22], broiler chicken traits [23] and laying traits [18]. A large number of SNPs in our resource population used 600 K Affymetrix Chicken SNP [24–27] have been discovered.

In the present study, we utilized two chicken lines to generate an F_2_ population. One of the lines was Single Comb White Leghorn (WL) and the other was Dongxiang Blue-Shelled Chicken (DX) with a single comb, both lines were selected for egg production. The WL has undergone intense artificial selection since the 20^th^ century and layers produce a higher number of eggs than female DX. The DX chicken is a Chinese indigenous breed, the onset of laying is later than in the WL [28]. The selection of fecundity indirectly leads to changes of comb characteristics [29], therefore, the WL and DX have a different comb weight. This cross enables us to detect underlying genetic differences that arise between the commercial and indigenous population. Here, we conducted a GWA analysis on the comb in a population of birds at 72 weeks old to document the associated genomic loci and genes that contribute to the comb. This research could be useful in understanding the physiological and genetic architecture of the chicken comb.

## Materials and Methods

### Ethics statement

This study was performed in strict accordance with Guidelines for Experimental Animals established by the Ministry of Science and Technology (Beijing, China). All protocols and procedures were approved by Institution Animal Care and Use Committee both in Poultry Institute, Chinese Academy of Agricultural Science, Yangzhou, China and College of Animal Science & Technology, China Agricultural University, Beijing, China.

### Study population and sample collection

An F_2_ resource population was generated by reciprocal crosses from a standard breed of White Leghorn and a Chinese indigenous strain of Dongxiang Blue-Shelled chickens. Detailed information on the source and management of the WL and DX strains and the basic characteristics of their intercross, have been described in previous papers [24–27, 30, 31]. For the F_0_ population, WL (6 ♂ 80 ♀) and DX (6 ♂ 133 ♀) were in the initial reciprocal cross, generating 1,029 and 552 chicks for the F_1_ population, respectively. Then, the F_2_ population in a single hatch, originating from 49 half-sib and 590 full-sib families, were produced from a WL/DX (25 ♂: 407♀) and DX/WL (24 ♂: 235♀) cross in the F_1_ generation. The F_2_ birds were bred indoors at the research base in Jiangsu Institute of Poultry Science, Yangzhou, China, under standardized conditions. The hens were placed in single-hen cages for laying and in a 16L: 8D lighting regime with feed and water *ad libitum* that met all NRC requirements. The F_2_ population were analyzed for various phenotypic traits, including egg weight, egg shell, residual feed intake, and yolk weight [18, 25, 26, 31]. After filtering the phenotypic information and verifying pedigree, 1482 hens from the F_2_ resource population were chosen for SNP genotyping. The SNP genotyping for each individual was performed by PLINK v1.90 program [32] after quality control and GWA analysis.

### Phenotypic data collection and analysis

All animals were humanely sacrificed by 60%–70% carbon dioxide at 72 weeks of age, the comb were taken from autopsy parallel to the head with fine scissors. Comb characters, including the length, height, and weight, were measured. The CL and CH were measured to the nearest 0.01 mm with a Vernier caliper. CL and CH were measured in accordance with the study by Eitan [33]. The CW was measured to the nearest 0.1 g on an electronic balance. After calculating all the bird combs at 72 weeks, a total F_2_ sample size of 1482 was employed.

Descriptive phenotypic statistics were calculated with the MEANS procedure of the SAS software package using all available records. To test normality and converse trait deviation to normality, the RANK procedure in SAS was utilized for rank-based inverse normal transformations (INTs).

### Genotyping and quality control

Blood samples were obtained using standard venipuncture. The genomic DNA was extracted from blood samples using the standard phenol/chloroform method and genotyped against a 600 K Affymetrix Axiom Chicken Genotyping Array (Affymetrix, Inc. Santa Clara, CA, USA). In order to carry out genotype calling and quality control (QC), the Affymetrix Power Tools v1.16.0 (APT) (http://affymetrix.com/) software with Axiom GT1 algorithm was then implemented. All cases of samples with dish quality control (DQC) ≤ 0.82 and call rate ≤ 97% were excluded in the downstream analyses. An R script adopted by Affymetrix was run to calculate the SNP QC metrics and filter out individual SNPs falling below given thresholds. After the application of APT for QC, 1512 individuals and 532,299 SNPs remained valid. To promote the effectiveness of the detecting quality the PLINK v1.90 program [32] was utilized for further QC. The SNPs with minor allele frequency (MAF) < 5% and Hardy-Weinberg equilibrium (HWE) test P < 1 × 10^−6^ were removed from subsequent analysis. Then BEAGLE v4.0 package was imputed for some sporadic missing genotypes [34], only SNPs with imputation quality score R^2^ > 0.5 were retained. After these steps, 1512 individuals and 435,867 SNPs were used in further GWA analysis.

### Genome-wide association analysis

Prior to GWA analysis, principal component analysis (PCA) was conducted in the PLINK package, because spurious associations could result from the presence of cryptic relatedness or hidden population stratification, a method of simpleM [35] was used to corrected the number of multiple tests for determining the thresholds for genome-wide significant/suggestive associations. Using this method, we obtained 59,308 suggested independent tests. Genome-wide significant and suggestive P-values were 8.43 × 10^−7^ (0.05/59,308) and 1.69 × 10^−5^ (1.00/59,308), respectively.

The GEMMA v0.94 package [36], referred to as a genome-wide efficient mixed-model association with an efficient exact mixed model approach, was implemented with the valid individuals and SNPs for univariate analysis. The method used, makes exact GWA analysis computationally practical for large sample sizes. Independent SNPs were used to compute the centered relatedness matrix, and the significance P-value level between SNPs and phenotypes was calculated from a derived Wald test. The model for GWA analysis was as follows:

y=Wα+xβ+u+ε

Where *y* represents a vector of phenotypic values for *n* individuals; *W* is a matrix of covariates (fixed effects with a column of 1s and top five PCs), *α* is a vector of the corresponding coefficients including the intercept; *x* is a vector of the genotypes of the SNP marker, *β* is the effect size of the marker; *u* is a vector of random individual effects; *ε* is a vector of random errors.

We used the “gap” packages [37] in the R project to draw the Manhattan plots and quantile—quantile (QQ) plots. Then the GenABEL package [38] in the R project was employed to calculate the genomic inflation factor, which was the judgement for the extent of false positive signals.

### Conditional and linkage disequilibrium analysis

Some SNPs have strong linkage to causal mutants, which leads them to be passively and significantly associated with target traits. Conditional analyses were performed by GEMMA, and linkage disequilibrium (LD) analysis was implemented by Haploview v4.2 [39]. In conditional analysis, the genotypes of one candidate gene are used as covariates until the p-value shows no GWA significance. In the analysis of Haploview v4.2, a block is produced using the solid spine algorithm, and defined as the first and last SNPs in a region with strong LD (D′ ≥ 0.8) with all intermediate SNPs. After these steps, the independent association signals in a putative region were distinguished.

### Estimation of variance explained

The heritability explained by the eligible SNPs (*h*^*2*^_*snp*_) for GWAS were estimated by the GCTA v1.24 program [40], which implements the method of univariate restricted maximum likelihood (REML). We also quantified the pair-wise phenotypic and genetic correlations for each character with the bivariate mixed model. A genetic relationship matrix (GRM) built from all genotyped SNPs on autosomes and two linkage groups, partitioned the chicken genome into 28 autosomes and two linkage groups, and jointly estimated their contributions to phenotypic variance (CPV) for traits [25].

The top five PCs produced by the GCTA program were chosen as covariates to estimate the variance contributed by each chromosome. The regression analysis was calculated by R to evaluate the relationship between the variance explained by each chromosome and its length. Besides this, we also estimated the CPV made by these associated loci or each chromosome after these associated loci were fitted as covariates.

### Gene identification and annotation

The annotated genes that were nearest or harboring significant SNPs were identified as candidate genes in which significant loci were located [41]. Genes in a specific genomic region [42] were detected by using BioMart tools and Variant Effect Predictor (VEP) based on the galGal4 assembly supported by Ensembl and NCBI annotation of the *Gallus gallus* genome version 4.0.

## Results

### Phenotypic description and genetic parameters

Means and standard deviations for comb characters, including CL, CH, and CW are presented in Table 1. After rank-based inverse normal transformation, all phenotypic values conformed to the normal distribution. The CW displayed largest coefficients of variation (44.48%), which was probably because the comb had not been chosen as a selection index. The additive genetic variation captured by all eligible GWAS markers associated with comb traits were quantified by univariate GCTA, the analyses found that all comb characters had highly heritable patterns (Table 2), and the highest SNP-based heritability estimate was found in CW (*h*^*2*^_*snp*_ = 0.69). Further, bivariate GCTA analyses revealed that comb traits are highly and positively interrelated. Each comb trait showed high genetic correlations (*r*_*g*_ = 0.76–0.94).

**Table 1:** Descriptive statistics for comb traits in the F_2_ population.

Trait^a^	Mean	SD	CV (%)	Min	Max
**CL (mm)**	35.82	3.26	9.11	19.84	47.42
**CH (mm)**	36.68	6.6	17.99	10.54	73.56
**CW (g)**	6.21	2.76	44.48	0.83	19.2

Abbreviations: Mean = arithmetic mean; SD = standard deviation; CV = coefficient of variance; Min = minimum; Max = maximum.

^a^CL = comb length; CH = comb height; CW = comb weight.

**Table 2:** Summary of genetic analysis for comb traits.

Trait^a^	CL	CH	CW
**CL**	0.61(0.041)	0.76(0.038)	0.81(0.029)
**CH**	0.60	0.61(0.040)	0.94(0.011)
**CW**	0.64	0.83	0.69(0.053)

Diagonal: heritability estimates. Lower triangle: phenotypic correlations. Upper triangle: genetic correlations. Standard errors of the estimates are in parentheses.

^a^CL = comb length; CH = comb height; CW = comb weight.

### Identification of candidate loci by GWAS

A total of 328 genome-wide significant associations were identified with CL, CH, and CW (Table 1). Almost all the significant locus on chromosome 1 (GGA1) were in a genomic region ranged from 167.7 to 179.8 Mb, and the significant loci on GGA4 showed differences among comb characters but were distributed from 0 to 2.9 Mb (Table 3). The detailed information for genome-wide significant SNPs are presented in S1 Table.

**Table 3:** Number and distribution of significant SNPs for comb traits.

Trait^a^	Chr^b^		Region	
	GGA^c^1	GGA4	GGA1	GGA4
**CL**	78	49	168.1–170.0M	0.0–1.6M
**CH**	12	185	169.2–169.8M	0.0–2.2M
**CW**	37	231	168.1–170.0M	0.0–2.9M
**total**	93	235	167.7–170.0M	0–2.9M
**Venn diagram**	11	49	169.2–169.8M	0–1.6M

Abbreviations:

^a^CL = comb length; CH = comb height; CW = comb weight.

^b^Chr = chromosome;

^c^*Gallus gallus* chromosome.

**Fig 1:**
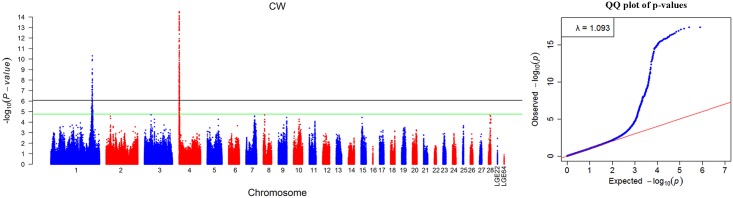
Manhattan plot (left) and quantile-quantile (QQ) plot (right) of the observed *P* values for the comb weight (CW). The Manhattan plot shows the -log_10_ (observed *P* values) for association of SNPs (y-axis) plotted against their chromosomal positions on each chromosome (x-axis), and the horizontal black and green lines depict the genome-wide significant (8.43 × 10^−7^) and suggestive significant (1.69 × 10^−5^) thresholds, respectively. For the QQ plot, the x-axis indicates the expected -log_10_-transformed *P* values, and the y-axis shows the observed -log_10_-transformed *P* values. The genomic inflation factors (λ) are shown on the top left in the QQ plots. Green points represent the genome-wide significant associations.

**Fig 2:**
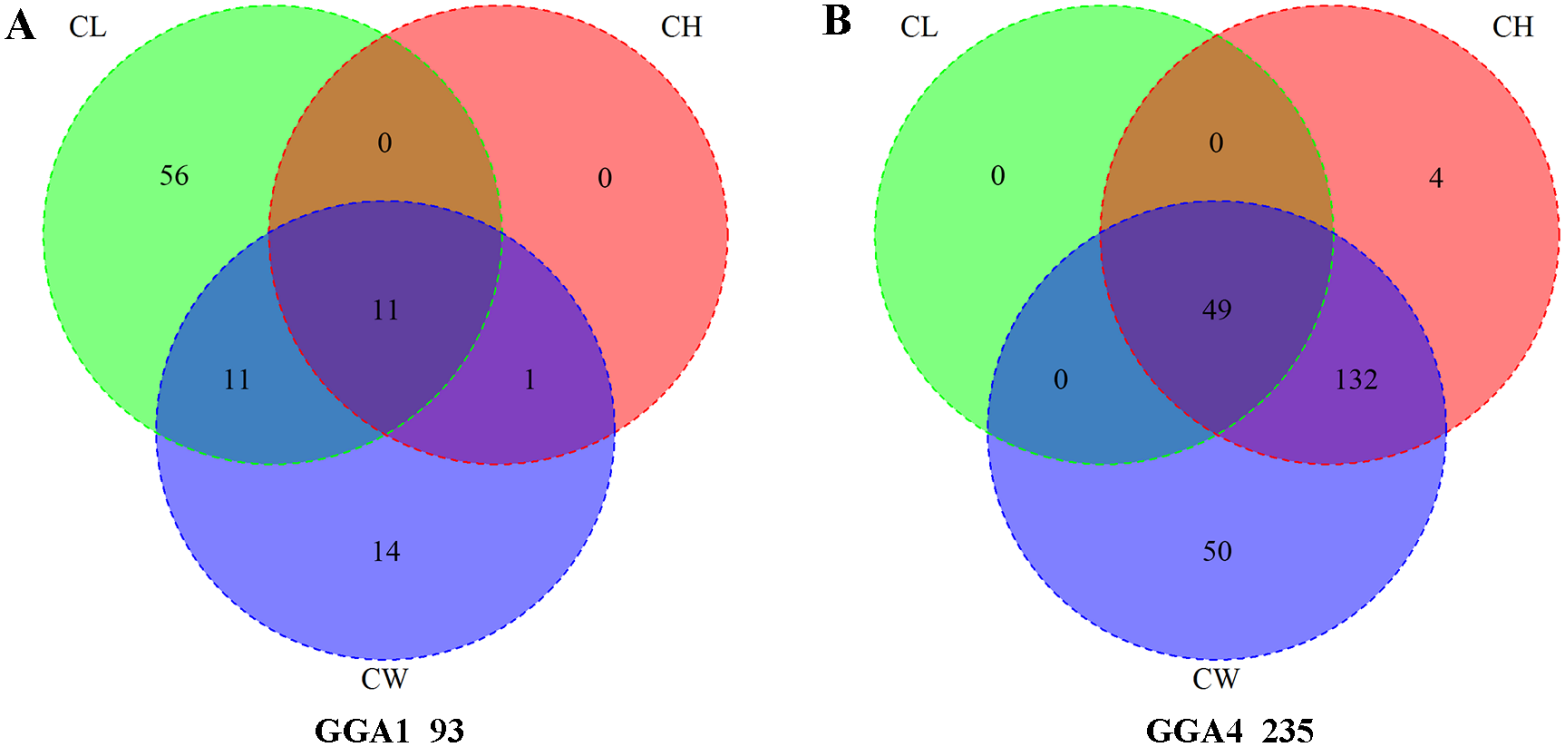
Venn diagram of significant SNPs on GGA1 (left) and GGA4 (right) associated with three comb characters by univariate association test. Comb length, comb height and comb weight are abbreviated as CL, CH, and CW, respectively.

The global view of the putative P-values about Manhattan and QQ plots for all SNPs affecting CW are given in Fig 1, and the remaining characters are shown in S1 and S2 Figs. The genome-wide discovery analyses yielded a small genomic inflation factor (λ) for each comb trait, ranging from 1.067 to 1.153. We then analyzed the significant locus by the Venn diagram, 60 SNPs out of these loci had a pervasive effect on CL, CH, and CW (Fig 2). Meanwhile, the candidate genes that nearest or harboring the SNPs are shown in S2 Table.

**Fig 3:**
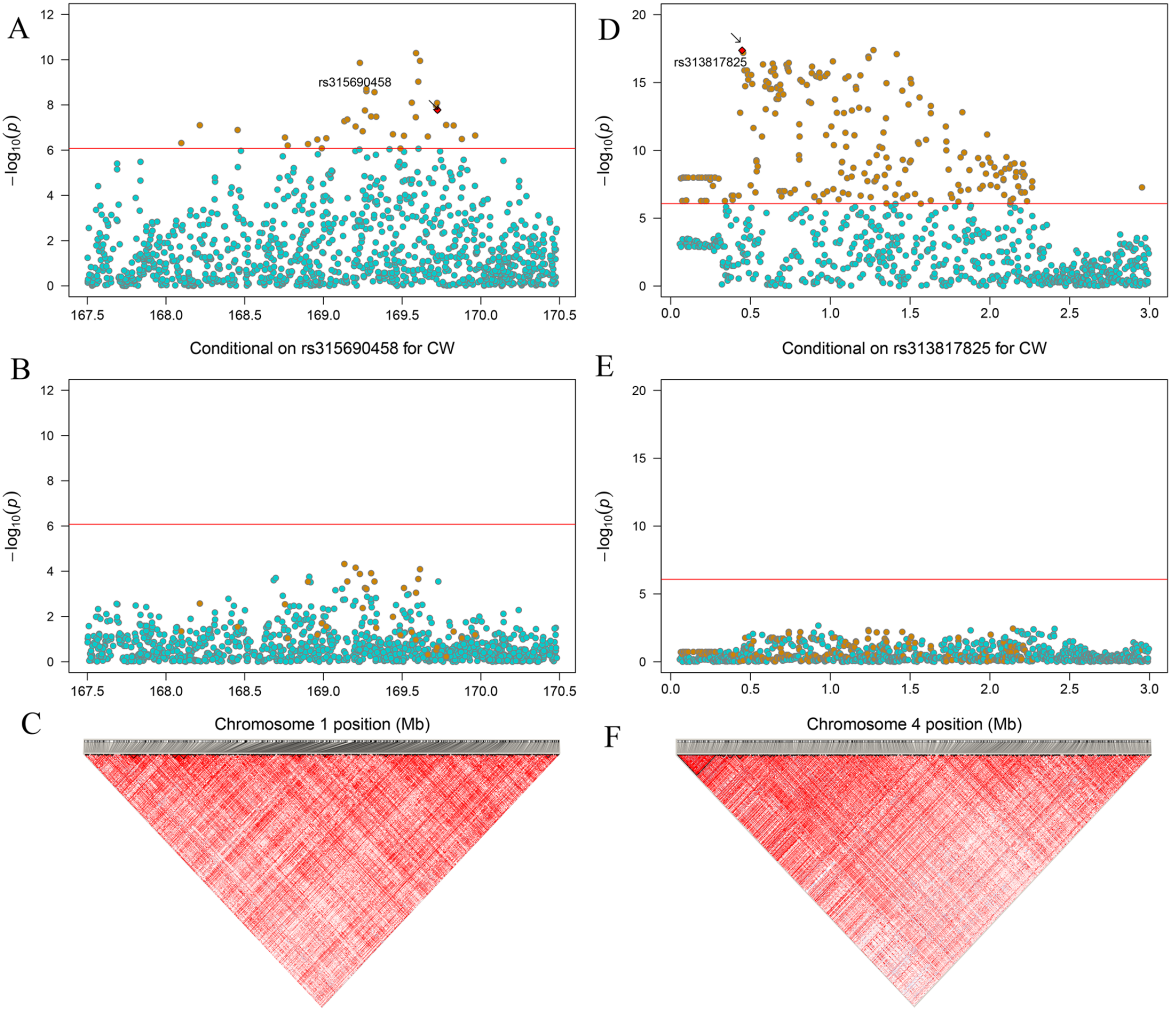
Regional plot for single nucleotide polymorphisms (SNPs) at GGA1 spanning from 167.5–170.5 Mb. **Plot A**: In the region 167.5 to 170.5 Mb the -log_10_ (observed *P* values) of the SNPs (y-axis) are presented according to their chromosomal positions (x-axis). Thirty-seven SNPs reached a genome-wide significance level (orange dot, 8.43 × 10^−7^). **Plot B**: The genotype of *rs315690458* was placed into the univariate test as covariance for conditional analysis. After conditioning on *rs315690458*, the significant SNPs in plot **A** (orange dot) were all substantially attenuated below genome-wide significant level in plot B. **Plot C**: Two hundred and seventeen small-scale blocks were observed in this region.

Since the putative variants may be in high LD with a causal locus genuinely associated with phenotype, the Haploview was implemented to infer the LD block. The results showed that the uncovered SNPs in GGA1 were in extremely strong LD status. Then stepwise conditional GWASs were performed to separately prioritize SNPs owing to the potentially strong LD between neighboring variants. After conditional analysis, the mentioned significant loci, *rs315690458* on GGA1 and *rs313817825* on GGA4, were found to be independent signals. Considering the above analysis, two significantly independent SNPs associated with all comb characters were analyzed further. Since *rs315690458* and *rs313817825* affected all comb characters, the regional association plots for CW were plotted to compare the difference of putative significance levels before and after the two loci (Fig 3) and the other characters for the regional plot are diagrammed in S3 and S4 Figs.

### Promising genes related to comb character

Gene annotation of significant SNPs will help us to find candidate genes related to comb characters. We scanned the significant region on the BioMart system of the two leading SNPs, *rs315690458* on GGA1 and *rs313817825* on GGA4, which both lie within introns of *VPS36* and *AR*, respectively (Table 4). Consequently, we first considered *VPS36* and *AR* as the primary candidate genes associated with the comb.

**Table 4:** Contributions of three mutations and genomic regions to comb characters.

SNP	rs315690458	rs313817825	rs314164847
**Chr**	1	4	4
**Position (bp)**	169,724,568	447,807	1,181,821
**Gene symbol**	*VPS36*	*AR*	*WNT11B*
**Location**	intron	intron	missense
**EA/AA**	C/T	C/T	G/A
**MAF**	0.483	0.495	0.477
**Amino acid change**	-	-	Glu/Lys
**CL**			
beta (SE)	-0.340(0.058)	-0.245(0.044)	-0.227(0.044)
CPV (%)	5.22	2.81	2.81
P-value	7.69E-09	2.38E-08	2.99E-07
**CH**			
beta (SE)	-0.308(0.059)	-0.346(0.043)	-0.288(0.044)
CPV (%)	4.30	5.36	5.51
P-value	2.12E-07	2.44E-15	6.20E-11
**CW**			
beta (SE)	-0.336(0.059)	-0.376(0.043)	-0.326(0.043)
CPV (%)	5.28	6.49	6.29
P-value	1.67E-08	4.31E-18	7.12E-14

Abbreviations: SNP, single nucleotide polymorphism; Chr, chromosome; EA, effect allele (minor allele); AA, alternative allele (major allele); MAF, minor allele frequency; CL, comb length; CH, comb height; CW, comb weight. Estimated allelic substitution effect per copy of the effect allele (EA); SE, standard error of the beta, which means the effect size of minor alleles; CPV, contribution to phenotypic variance (%).

For SNP-trait association analysis, further utilizing gene annotation of the causal locus allowed us to screen the putative genes relating to comb character, and the missense mutations on exons were more meaningful [43]. We then totally identified one missense locus *rs314164847* at GGA4 on gene *WNT11B* (wingless-type MMTV integration site family, member 11b), and considered *WNT11B* as a third candidate gene.

### Allelic contribution to phenotypic variation

**Fig 4:**
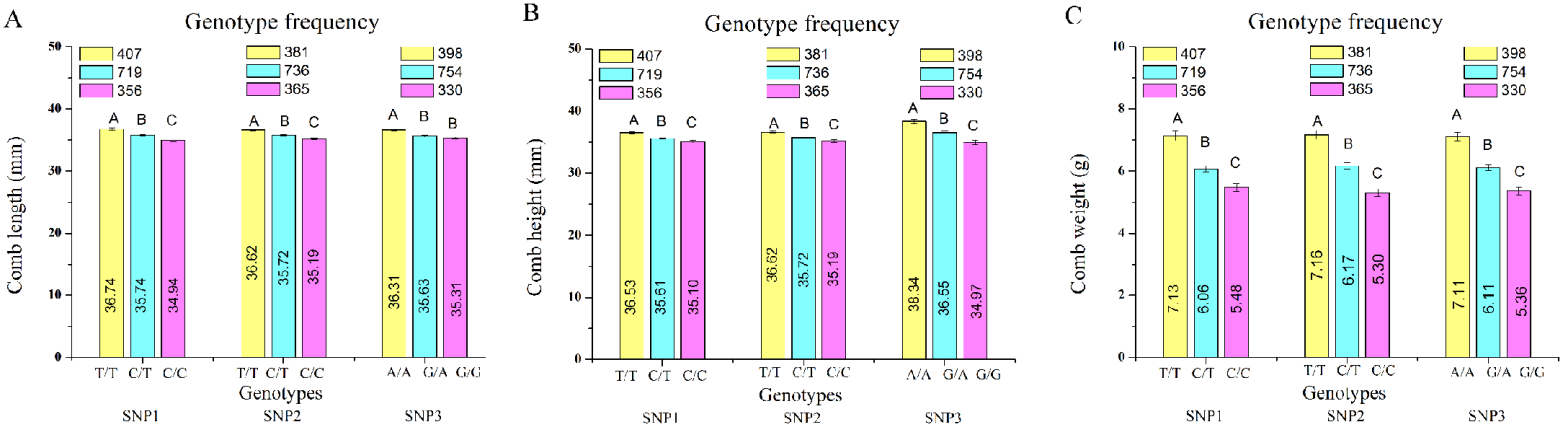
Genotype effect plot of three leading SNPs for comb characters. SNP1 = *rs315690458*, SNP2 = *rs313817825*, and SNP3 = *rs314164847*. Phenotypic differences contributed by the three loci of SNP1, SNP2, and SNP3 on genes of *VPS36*, *AR*, and *WNT11B*. Plots A, B, and C describe the phenotypes of CL, CH, and CW among three genotypes at SNP1, SNP2, and SNP3, respectively. The yellow, blue and purple bars represent major-allele homozygotes, heterozygotes, and minor-allele homozygotes, respectively. The number of samples for each genotype is indicated at the top.

Real phenotype frequencies between three genotypes at each locus were compared. These showed that the three phenotypes revealed significant segregation (Fig 4). The allelic substitution effects and phenotypic variance explained by them were estimated for all comb characters (Table 4). The MAF at each locus is treated as the effect allele according to the GEMMA definition. The results suggested that the heterozygote of the effect allele possessed medium characteristics between the two homozygotes, revealing that individual phenotype was more severely affected by homozygotes. Mutations from C to T at locus *rs315690458* caused all the comb characters to increase. The major allele of *rs315690458* was favorable for a larger comb, with a phenotypic difference of 5.15%–30.10% between opposite homozygous genotypes (S3 Table), which explained over 4% of the phenotypic variance for comb characters. A mutation from C to T at locus *rs313817825* also causes all comb characters to decrease, and a phenotypic difference of 3.91%–35.09% between the two homozygous genotypes, the contribution phenotypic variance ranged from 2.81% to 6.49%. Specially, *rs313817825* revealed the largest phenotypic variation explained for CW.

### Genome partitioning of genetic variation

**Fig 5:**
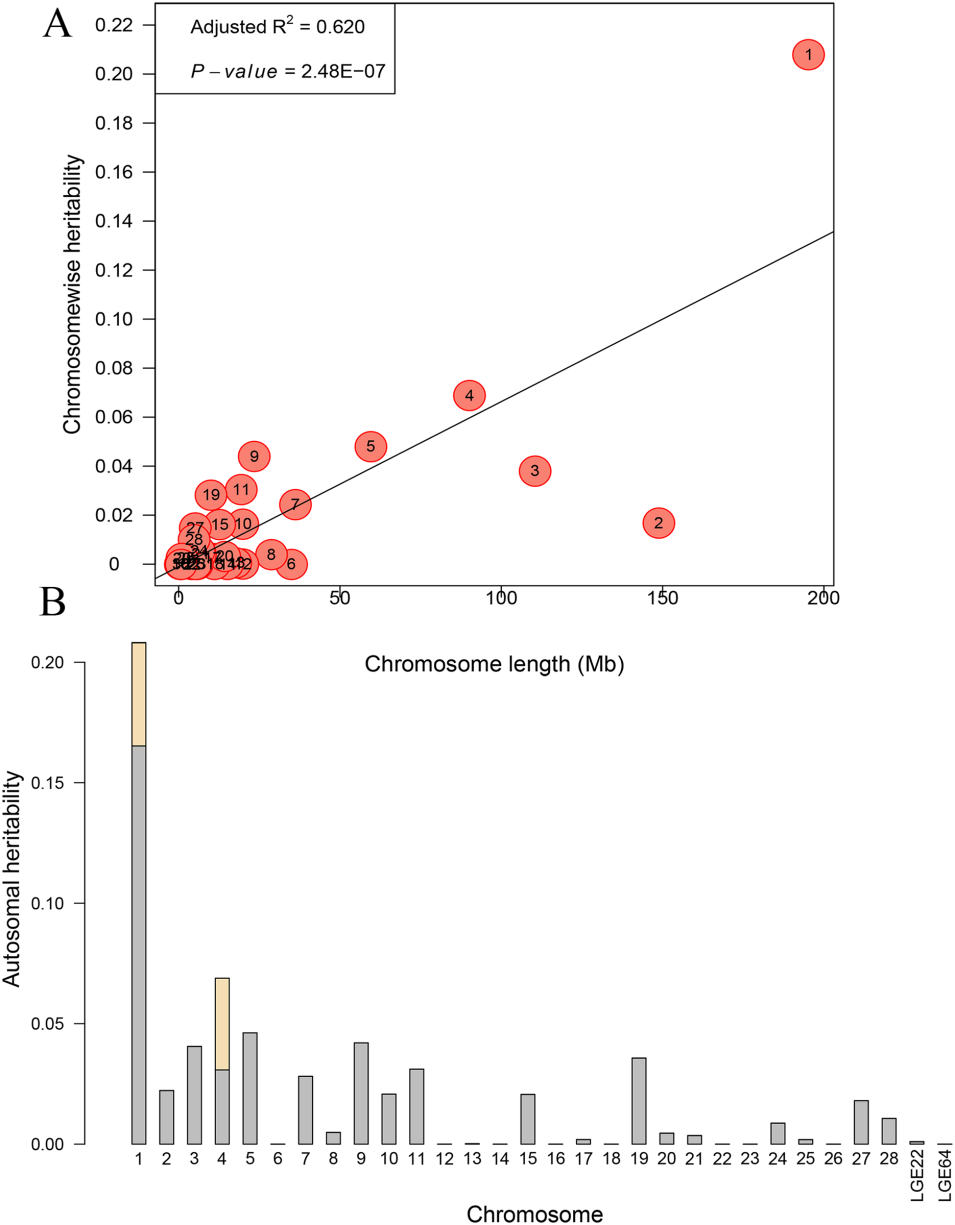
Genome partitioning for comb length by joint analysis. **A.** The estimated proportion of variance captured by each chromosome against its size. The characters in the circles are the chromosome numbers. **B.** Contributions of genome-wide association study (GWAS) SNPs partitioned by chromosome. The whole bars indicate the estimates of variance explained by each chromosome, in which the two wheat bars represent the same values by three resulting loci.

We implemented an exploratory analysis through partitioning the genetic variation onto chromosome segments to further illustrate the genetic architecture of comb characters. Only the partitioning spectrum of CL could be estimated, for the relatively small sample size in the F_2_ population parameter estimates for CH and CW in the joint model could not converge. The estimates of variance contributed by each chromosome exhibited a strong linear relationship with the length of the chromosome for CL (R^2^ = 0.620, Fig 5A), for GGA1 explained 20.80%, and GGA4 explained 6.89% of phenotypic variance. To quantify the effects of the two resulting variants on CL, we fitted the two leading SNPs as covariates and repeated the genome partitioning analysis. When compared with the results before adjustment, we found that the variance explained by GGA1 dropped to 16.53% (Fig 5B). The same estimate for GGA4 showed the largest decrease from 6.89% to 3.08%, and the estimates for the other 28 chromosomes almost remained the same.

## Discussion

The detection of QTLs associated with comb by utilizing the resource population at a genome-wide level has been limited in previous studies [16, 44]. Our F_2_ resource population, consisting of 1512 hens, is the largest and the first population used for comb GWA analysis so far, and was the first GWA analysis by higher density (600 K) SNP array covering chromosomes 1–28. Therefore, the results imply that the novel genomic region and locus identified by the current study may further contribute to studies of the comb. The heritability of the comb trait in our results was revealed to be higher than in other studies [9, 45], which suggested that the potential for selection of this trait will create greater genetic progress.

To further decipher the heritable architecture in CL, we partitioned the genetic variance onto different chromosomes based on the estimated chromosomal GRMs. A strong linear correlation between the variance explained by each chromosome and its length were observed in our study, which is consistent with previous findings [25, 46]. We found population stratification in all GWAS analyses of comb. In practice, it is usually considered that a genomic control inflation factor (λ) less than 1.05 indicates no population stratification [47]. The genomic inflation was caused by a large number of associated SNPs [18] and the results in our study showed that we have an abundance of significantly associated SNPs. Above all, comb may be expected to be under polygenic architecture [21]. Specially, GGA1 accounted for the largest genetic variance (20.80%), may due to GGA1 accounting for 14.9% of the entire genome [48]. GGA4 accounted for 6.86% of the genetic variance, while the genetic variance dropped to 3.08% after the leading SNP *rs313817825* was fitted as a covariate. Genome partitioning analysis revealed that the contribution of phenotype variance by *rs313817825* (2.81%) accounted for nearly half of the whole GGA4 genetic variance. The largest phenotypic variation explained by *rs313817825* was for CW. The *beta* effects of SNPs for all comb traits were negative, which indicated the allele resulting in the lower CL, CH, and CW, and hens with the opposite homozygote alleles showed the highest CL, CH, and CW.

Our results found that causal genomic regions or genes controlling comb characters were discovered on GGA1 and GGA4. A previous study of QTL affecting CW was detected on GGA1 and GGA3 by Johnsson et al. [16] and another study by Podisi [49] showed QTL on GGA4, GGA5, and GGA9. The difference between the present study and previous reports might be explained by the different populations used and different ages when comb measurements were taken. However, these studies utilized QTL analysis and a limited population. For our study, since both phenotypic correlation and genetic correlation were high, and the chromosome regions for comb were close, it is reasonable to speculate that the same SNPs affected these traits.

After step-wise GWA analysis, Venn diagram analysis, LD and conditional analysis, three loci on GGA1 and GGA4 showing significant GWA with comb characters were detected, these were considered the most important putative variants. The associated region on the GGA1, from 168.1–170.0 Mb, significantly influenced the growth trait [50]. Among potential candidate genes are *WDFY2* (WD Repeat And FYVE Domain Containing 2), *CKAP2* (cytoskeleton associated protein 2), *RNASEH2B* (ribonuclease H2, subunit B), *ATP7B* (ATPase, Cu++ transporting, beta polypeptide), *LOC428073* (fibrinogen-like protein 1-like) and *VPS36* (vacuolar protein sorting 36), which have important effects on growth. *CKAP2* modulates cell survival [51]. *RNASEH2B* was known to specifically downgrade RNA [52]. So far, no association has been found for the genes above with comb traits in chickens.

*VPS36* plays an important role in the ESCRT (endosomal sorting complex required for transport) pathway [53] and is one of the important endocytosis components that activates growth factors, hormones and cytokine receptors inside the cell and delivers them to lysosomes [54]. After step-wise analysis, the locus *rs315690458* located on *VPS36* was considered the most significant SNP on GGA1.

Previous studies showed that *VPS36* mRNA was detected in both granulosa and theca layers, especially in the prehierarchical follicles, which is likely to be involved in the process of ovarian follicular development [55], and is regulated by *FSH* and estradiol in chicken follicles [56]. *VPS36* mRNA displayed a gradual decrease with the increasing concentration of estrogen. Sun et al [26] revealed that the region from 169.01 to 169.7 Mb was also related to follicle weight. Considering the function of *VPS36* mentioned above and the comb involvement in heat regulation [2] in the chicken, while puberty turns to sexual maturity the comb reddens. Studies have shown that larger comb could be used as an indicator of fecundity [15] with higher reproductive capacity [4]. Gene *VPS36* may participate in comb growth, however its involvement is not clear.

On GGA4, in the range 0–2.9 Mb, genes include *AR* (androgen receptor), *WNT11B* (wingless-type MMTV integration site family, member 11B), *EFNB1* (ephrin-B1), *FAM155*B (family with sequence similarity 155, member B), *IGBP*1 (immunoglobulin binding protein 1), *PDZD1*1 (PDZ domain containing 11), *TAF9* (TAF9 RNA polymerase II), and so on. *EFNB1* may play a role in cell adhesion and function in the development or maintenance of the nervous system [57]. *IGBP*1 is related to proliferation and differentiation of B cells [58].

In our study, the two loci, *rs313817825* and *rs314164847*, located on AR and WNT11B showed a significant association with the comb. AR is a nuclear hormone receptor of the NR3C class [59, 60], which is involved in the development of primary and secondary male sexual characteristics, maintenance of sexual function and possibly has a causative role in aggressive behavior [61]. It also has been suggested that the *AR* gene has numerous effects on reproductive and second sexual ornaments in chickens. Previous reports reveal that *AR* has been localized immunocytochemically in the chicken comb [62]. In large bodied males, the small amounts of androgen present in the plasma prior to 11 days after hatching would appear to be below the threshold for stimulation of comb and testis size [63]. Androgen’s actions are mediated by *AR* controlling the growth of the comb [64] and involved in the male reproductive system, stimulating testis and comb growth [65]. While stimulated with exogenous testosterone and dihydrotestosterone, the comb grows larger [66].

For female chickens, as the pullets advance into sexual maturity, the comb begins to get larger, and plasma estrogen level increases [8]. After sexual maturation, the comb will be fully developed by sex hormones, such as androgen, estrogen and testosterone [33]. While hens are laying, the comb begins to redden and with loss of comb redness ovarian regression occurs [67]. All the above evidences demonstrate that *AR* could be a crucial and promising candidate gene relating to comb growth and reproduction.

Another SNP, *rs314164847*, displayed significant association with the comb. It is located on gene *WNT11B*, a starting protein of the Wnt pathway, regulating organ morphogenesis [68]. *WNT11B* plays an important role in dermal development [69]. Wnt signaling is involved in embryonic muscle development and maintenance of skeletal muscle homeostasis in the adult [70], and the Wnt pathway plays an important role in human follicle morphogenesis [71] by repairing the epidermis during wound healing. The comb is composed of the epidermis, dermis and central layers [1], and contains a lot of blood vessels. In the current study, the *WNT11B* gene was identified with the comb, and is suspected to have a role in the process of comb growth, further investigations are needed.

Our study demonstrates the features and the genetic architecture present in animals. First, it highlights the importance of pleiotropic effects and linkage disequilibrium with QTL [16]. Although, we obtained candidate genes involving comb growth by rigorous statistical analysis, the actual significant region is located discreetly on GGA1 and GGA4. The results in this study and present research using the same population, reveal that the significant region on GGA1 is also associated with egg weight, ovary weight, and feed intake [18, 25, 26]. In other populations, it has been found that this region is also related to body weight [50, 52]. Most poultry breeders focus on body weight and fecundity, resulting indirectly in selection for comb character since the body mass significantly affected comb weight [29] and the comb is an indicator of sexual maturity [1]. The significant regions were detected in GWA studies through no or limited selection for comb traits, pleiotropic effects of the locus on other traits that are under selection, or close linkage and linkage disequilibrium with QTL that are under selection [72]. Second, this has important ramifications for understanding the genetic architecture underlying quantitative and qualitative traits. Different comb type are affected by copy number expansion or inversion that result in ectopic expression [73,74] in the mesenchyme of the developing comb region of the chicken embryo, while the significant regions in our study may determine the weight of the comb given the comb type formed. The qualitative trait was controlled by a major gene whilst the quantitative trait was controlled by polygenes.

## Conclusions

Our current study, is the first GWA analysis on comb characters in chickens. Our study provides evidence that the comb appears to be highly heritable and there is a high genetic correlation with each comb character. A total of 127, 197, and 268 genome wide significant SNPs for CL, CH, and CW, respectively, were found by GWA analysis with a 600 K high-density SNP array. The significant regions were 167.7–169.9 Mb on GGA1 and 0–2.9 Mb on GGA4. LD and conditional analysis suggested these regions were in extremely strong linkage disequilibrium status. A list of candidate genes *VPS36*, *AR*, and *WNT11B* were detected for their plausible function in comb characters. Considering the reliability of GWA analysis for the significant SNPs and its contribution to phenotypic variance, our findings establish a better understanding of the molecular controls involved in the development of the comb.

## Supporting Information

S1 Fig Manhattan plot (left) and quantile-quantile plot (right) of the observed *P* values for comb length (CL). (TIF)

S2 Fig Manhattan plot (left) and quantile-quantile plot (right) of the observed *P* values for comb height (CH). (TIF)

S3 Fig Regional plot for single nucleotide polymorphisms (SNPs) at GGA1 spanning from 167.5–170.5 Mb for comb length (CL) and comb height (CH), respectively. (TIF)

S4 Fig Regional plot for single nucleotide polymorphisms (SNPs) at GGA4 spanning from 0–3 Mb for comb length (CL) and comb height (CH), respectively. (TIF)

S1 Table Genome-wide significant SNPs for comb length (CL), comb height (CH), and comb weight (CW) by univariate model. (XLS)

S2 Table The genes harboring or being near SNPs that are significantly associated with all comb characters studied. (XLS)

S3 Table Genotype effects of three leading SNPs for comb length, height, and weight, respectively. (XLS)
