# Supplementary figures and images for: Genome-Wide Association Studies for Comb Traits in Chickens
Source: PLoS One. 2016 Jul 18;11(7):e0159081. doi: 10.1371/journal.pone.0159081 (PMC4948856; doi:10.1371/journal.pone.0159081)

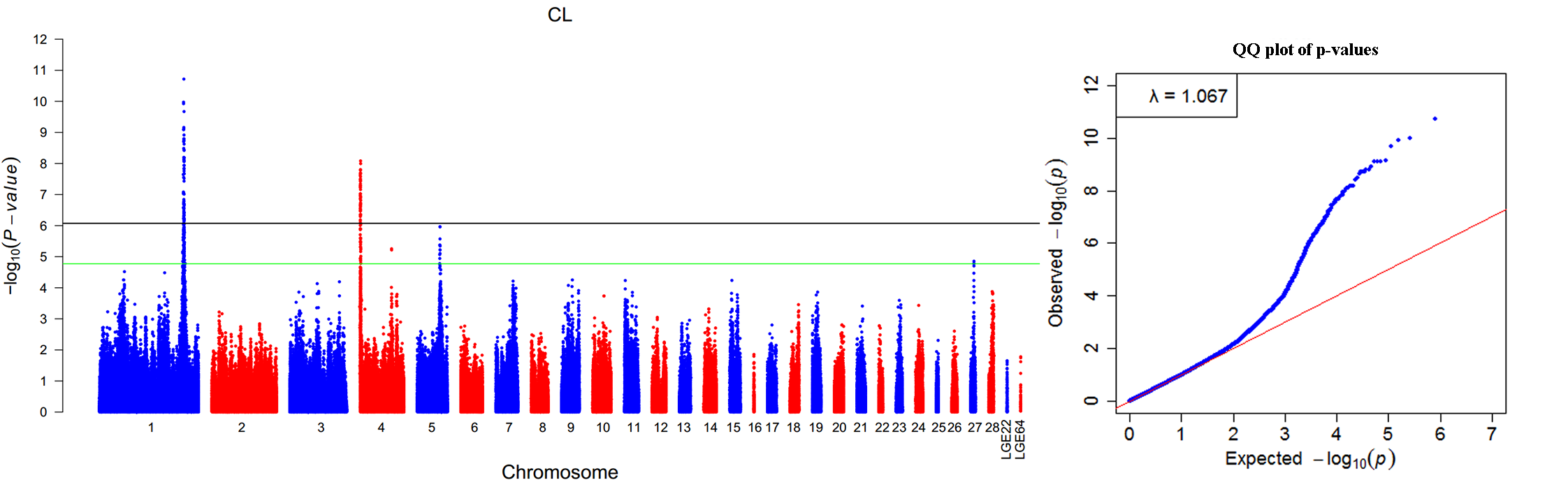

Supplement: S1 Fig — (TIF) [file pone.0159081.s001.tif]

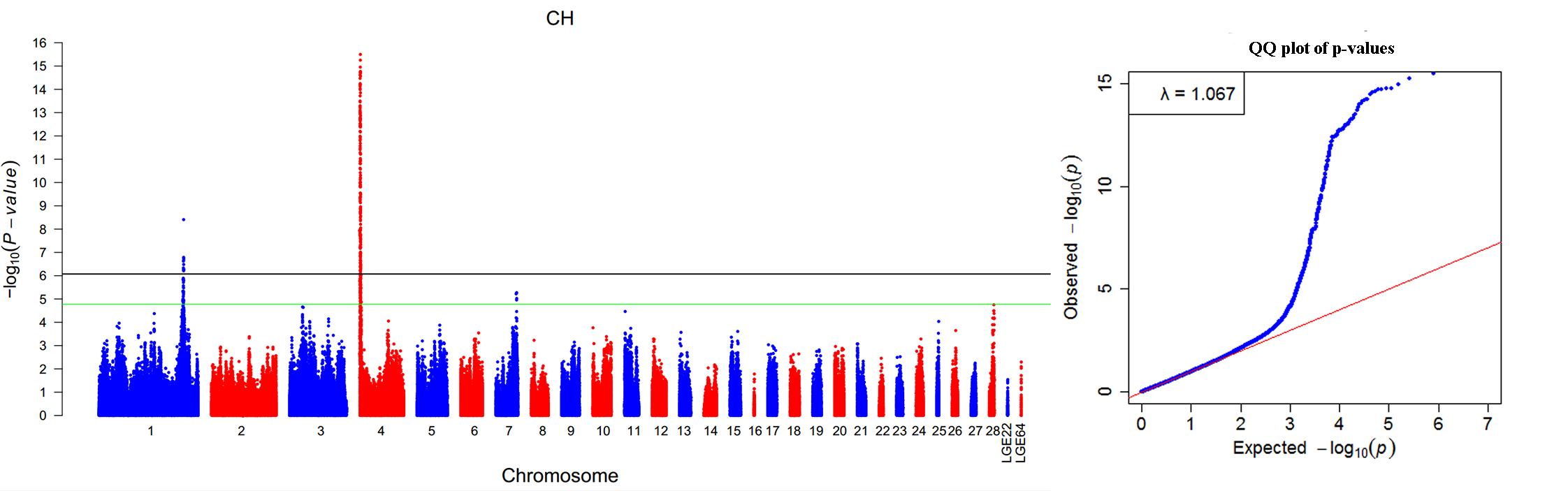

Supplement: S2 Fig — (TIF) [file pone.0159081.s002.tif]

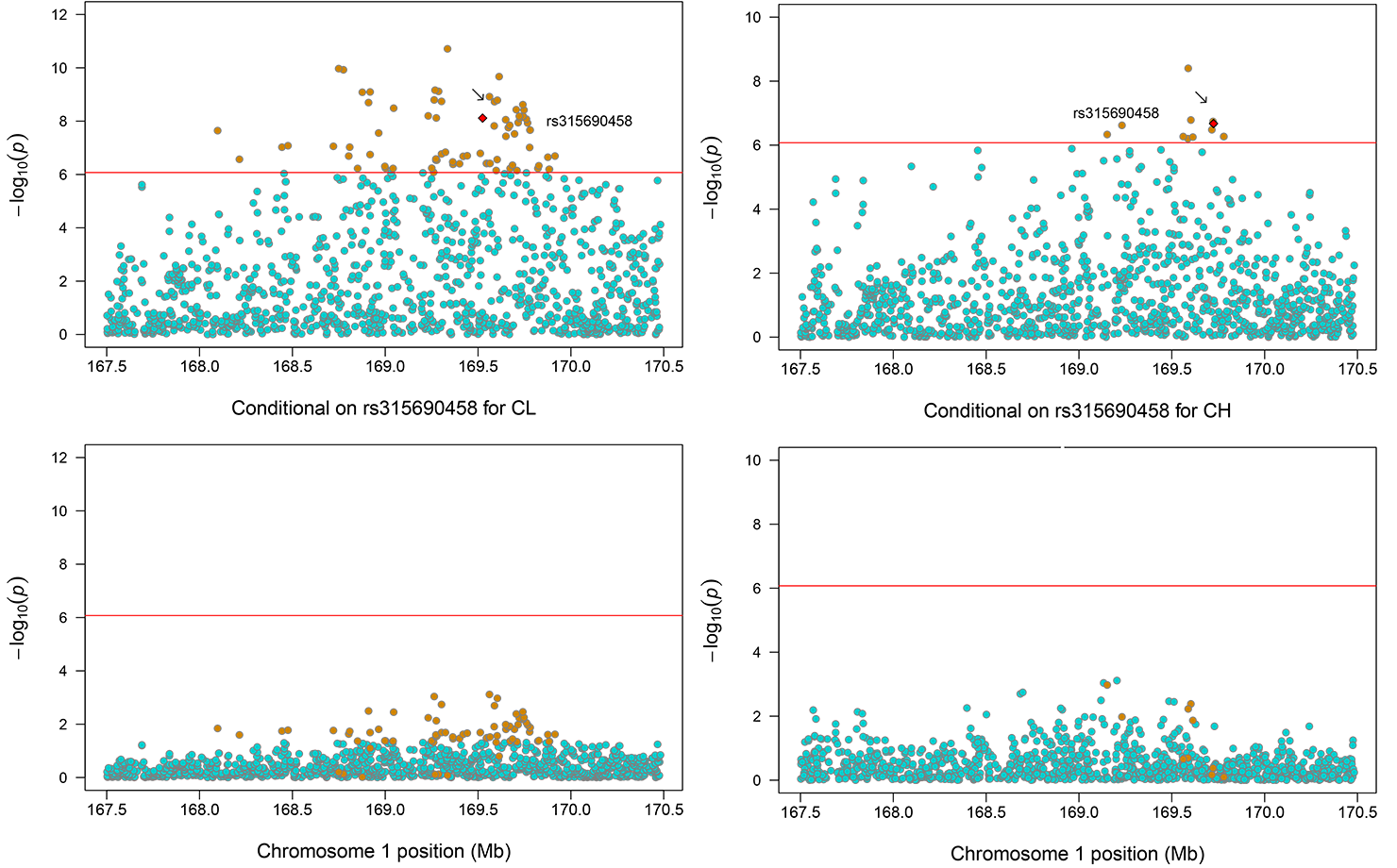

Supplement: S3 Fig — (TIF) [file pone.0159081.s003.tif]

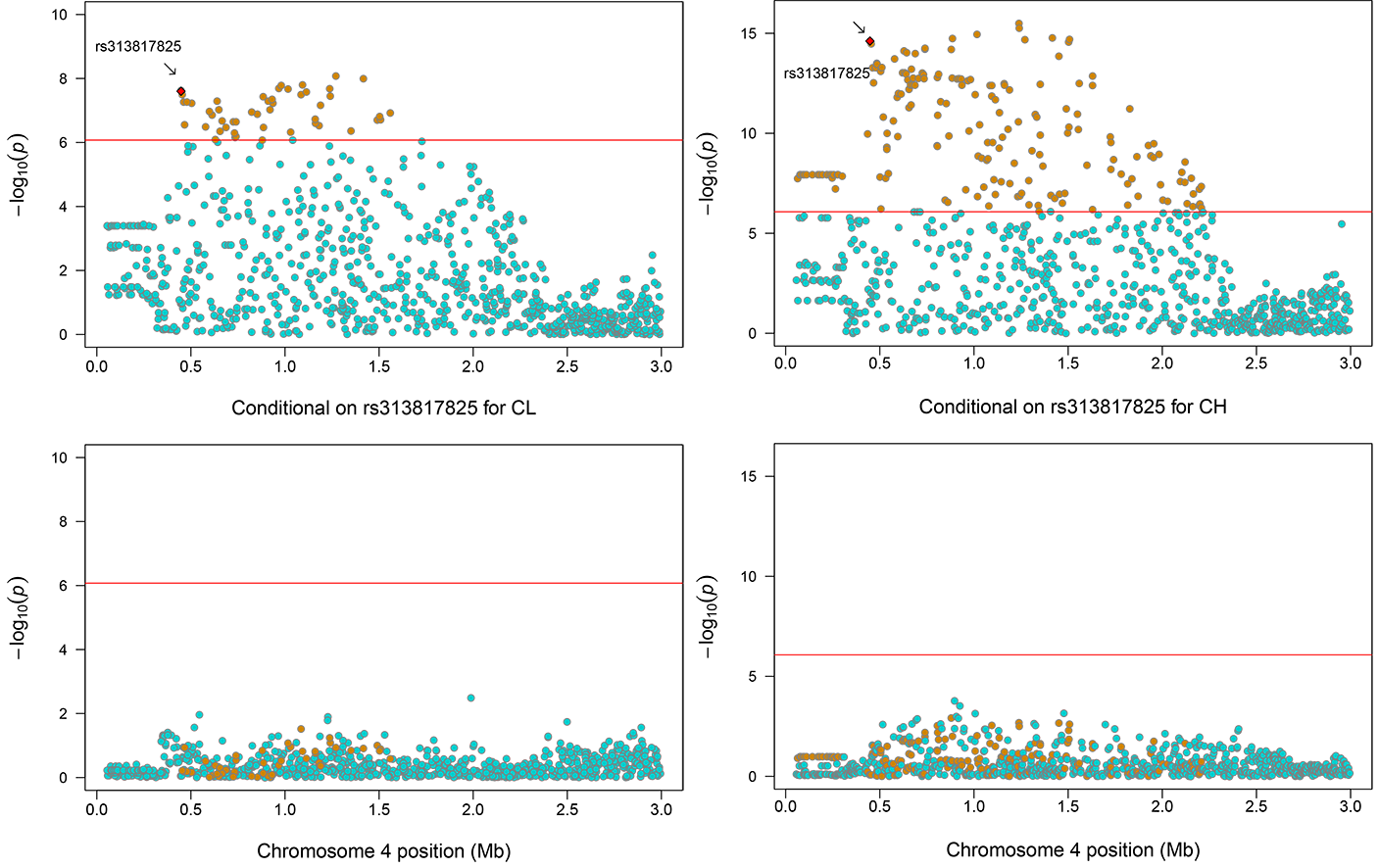

Supplement: S4 Fig — (TIF) [file pone.0159081.s004.tif]
